# Presidents and vaccines: head of state inoculation as a tool for vaccine promotion

**DOI:** 10.3389/fpubh.2024.1364927

**Published:** 2024-05-14

**Authors:** Lara Collart, Elie Lunanga, Nik Stoop, Marijke Verpoorten

**Affiliations:** ^1^Institute of Development Policy, University of Antwerp, Antwerp, Belgium; ^2^FWO – Research Foundation Flanders, Brussels, Belgium; ^3^Centre d’Expertise en Gestion Minière, Université Catholique de Bukavu, Bukavu, Democratic Republic of Congo; ^4^Centre for Institutions and Economic Performance, University of Leuven, Leuven, Belgium

**Keywords:** immunization, vaccine hesitancy, institutional trust, public health, Democratic Republic of Congo

## Abstract

**Introduction:**

Vaccine hesitancy, an important threat to global health, has increased since the onset of the COVID-19 pandemic. The public vaccination of high-profile figures, such as heads of state, has been touted as a potential tool for increasing vaccine acceptance among the general population. However, systematic information on such role modelling is lacking and existing studies focus on a small number of high-income countries. We take advantage of the COVID-19 pandemic to fill this gap.

**Methods:**

Through a systematic search of internet sources, we first document that most global leaders supported the vaccination campaign and actively communicated their vaccination status to the public. We then turn to a case study to provide experimental evidence on vaccine role modelling for a country in Africa – the region that is most lagging behind in achieving universal immunization coverage. We rely on a randomized survey experiment with 600 citizens in the Democratic Republic of Congo and take advantage of the fact that the Congolese President publicly received a COVID-19 vaccine during the survey period.

**Results and discussion:**

Our findings demonstrate that the impact of political leader’s role modelling is moderated by trust and depends on media outreach and access. When trust in leaders is lacking, or news on their actions is inaccessible, alternative ambassadors and effective communication methods become crucial in motivating and informing the public. This may be especially relevant in fragile states and remote regions.

## 1 Introduction

Leaders as diverse as United States President Joe Biden and Iran’s Supreme Leader Ayatollah Ali Khamenei have come forward on television to receive a COVID-19 vaccine. The idea is that, by getting vaccinated publicly, leaders signal that they are confident in the vaccine’s effectiveness and safety, thereby promoting vaccine acceptance among the broader population. Other heads of state, including Germany’s Angela Merkel and France’s Emmanuel Macron have revealed that they got vaccinated, but did not publicize the moment on television or with a picture. A small minority of heads of state publicly refused to get vaccinated. What is the relative frequency of these choices? And, to what extent do these leaders’ choices influence vaccine acceptance? These are the questions we address.

First, we create a public database of heads of state with systematic information on their support for COVID-19 vaccination, whether they are vaccinated themselves, and whether they distributed images of the inoculation. We find that 168 out of 173 global leaders (97%) explicitly supported the vaccination campaign. Most of them (80%) also made public that they received a COVID-19 vaccine, and 78% of those vaccinated publicized the news with a picture or video. We can therefore conclude that most global leaders thought it was important to communicate their vaccination status to the public using more than words.

Existing studies suggest that role modelling by political leaders helps to promote vaccine acceptance among the population. However, few studies support this with experimental evidence, and most focus on a small number of high-income countries. This study aims to fill this gap by providing experimental evidence on vaccine role modelling for a country in Africa – the region that is most lagging behind in achieving universal immunization coverage (1). We turn to the Democratic Republic of Congo, a country that has been particularly affected by declining vaccine confidence during the COVID-19 pandemic (2).

We conducted a survey with 600 Congolese citizens. Through a randomized survey experiment, 1/3 of respondents was prompted to consider the hypothetical vaccination of their president, while another 1/3 of respondents was prompted to consider the hypothetical vaccination of the Congolese Cardinal (of the Catholic Church). We compare their stated willingness to accept a COVID-19 vaccine to that of a control group who did not receive such prompt. While the survey was ongoing, president Tshisekedi publicly received a COVID-19 vaccine. We compare stated vaccine acceptance of respondents interviewed before and after Tshisekedi’s vaccination. Our analyses rely on multivariable logistic regressions controlling for respondent and household characteristics, and we formally assess the influence of potentially confounding unobserved characteristics.

While the hypothetical vaccination of the Cardinal had no significant impact on vaccine acceptance, the results for the president were moderated by public trust. For Congolese who report trusting the president, the survey experiment boosted acceptance from 27 to 52%. For those who mistrust the president, it decreased acceptance from 17 to 11%. When the president got vaccinated during the survey period, vaccine acceptance increased from 15 to 35%, but only for respondents who were aware of the president’s vaccination. These findings demonstrate that the impact of political leader’s role modelling is moderated by trust and depends on media outreach and access. When trust in leaders is lacking, or news on their actions is inaccessible, alternative ambassadors and effective communication methods become crucial in motivating and informing the public. This may be especially relevant in fragile states and remote regions.

In what follows, we first situate our contribution in the literature on vaccine hesitancy. We then present our database on vaccine role modelling of global leaders. Section 4 describes the context in which our case study took place. Section 5 presents our data and methods, while results are presented in Section 6. We conclude with a discussion of our findings in Section 7.

## 2 Vaccine hesitancy: causes and remedies

The World Health Organization (WHO) defines vaccine hesitancy as a “delay in acceptance or refusal of safe vaccines despite availability of vaccine services” (3). A growing body of evidence links vaccine hesitancy to demographic factors (such as gender and age), socioeconomic factors (including educational attainment and ethnic origin), as well as citizen’s perceived efficacy and safety of vaccines, which in their turn depend on previous vaccination history, (mis)information, and levels of trust in public authorities [e.g., (4–7)]. Given these determinants, it is unsurprising that vaccine hesitancy varies substantially, not only across countries, but also within countries, across different subsets of the population (8). Regarding COVID-19 vaccination, for instance, Solís Arce et al. (9) documented a wide cross-country variation in vaccine acceptance ranging from 30 percent in Russia to 97 percent in Nepal, but also large disparities within countries, such as an 18 percentage point difference between United States respondents who continued studies after secondary school and those who did not.

Importantly, vaccine-hesitant individuals may refuse some vaccines, but agree to others. The above-mentioned determinants may thus relate to the characteristics of a specific vaccine or vaccination process (3). In the case of the COVID-19 pandemic, factors that played a role in vaccine hesitancy included the many asymptomatic cases of COVID-19 which fed the idea of a rather harmless disease, the urgency surrounding the vaccine development which led some to doubt the reliability of clinical trials, and the social and economic disruption associated with the pandemic which turned out to be fertile ground for conspiracy theories (10–12).

In (2019), the WHO listed vaccine hesitancy among the main threats to global health (2023). Since then, COVID-19 caused a severe regress in global vaccination coverage and a sharp decrease in vaccine confidence (2, 15). To turn the tide and restore immunization progress, WHO, UNICEF, and other health partners announced “The Big Catch-Up” during the World Immunization Week (16). Through targeted efforts, the organizations aim to strengthen health care workforces, improve health service delivery and “build trust and demand for vaccines within communities” (16).

Scholars have argued that vaccine demand needs to be actively promoted by comprehensive communication campaigns to improve the perceived efficacy and safety of vaccines (17, 18). While there is a large body of evidence on what and how to communicate [see, e.g., (19–25)], less is known about who should communicate to reach maximum impact, and existing studies mainly focus on the United States [e.g., (26–28)].

Pioneering work, carried out across six countries, distinguished between the impact of COVID-19-related social distancing messages delivered by a well-known medical expert, a government official, a Hollywood actor, or a social media celebrity (29). The message had the largest impact on respondents’ stated intentions when delivered by the health expert, followed by the government official, who outperformed celebrities. The authors argue that, while celebrities have been shown to influence opinions about health and well-being at large, during times of crisis, health experts and government officials – who manage the crisis and are held accountable for it – may exert greater influence on public opinion.

The most prominent government official is arguably the head of state. Heads of state can influence citizen’s life and attitudes, not only by implementing policies, but also by communicating with the public, both with words and symbolic actions (30). This is in line with the social identity model of leadership, according to which leaders do not simply *represent* citizens’ attitudes and opinions, but can also *change* those, be it only for the subset of citizens that perceives the leader as part of the ‘ingroup’ (31, 32). That both words and actions by heads of state can have tremendous impact on crisis management, both positive and negative, has been amply demonstrated in the COVID-19 crisis. Both Brazil’s Jair Bolsonaro and USA’s Donald Trump, for instance, have aggravated the health crisis by downplaying the health risk of COVID-19, opposing measures to prevent its spread, and instead promoting remedies known to be ineffective (32–34). Conversely, several heads of state, including prime ministers Jacinda Ardern of New Zealand and Sanna Marin of Finland, have been credited with a better-than-average management of the health crisis (35).

One low-cost action that a head of state can take is to get vaccinated publicly. With this action, political leaders can arguably signal to the public that vaccines are safe and effective, thereby building public confidence in vaccines (18). Recent (quasi-)experimental research from a small number of high-income countries shows that citizens often follow cues from their political party’s elites, including when it comes to COVID-19 vaccination intentions (26–28, 36). For instance, while President Trump was mostly known for having anti-vaccination attitudes, he did receive a COVID-19 vaccine and, in one interview on Fox News, recommended citizens to get vaccinated as well. Levering this video in an online randomized experiment with millions of YouTube users, Larsen et al. (27) found that it significantly increased vaccination rates in treated United States counties.

We aim to contribute to this literature by systematically documenting the vaccine role modelling behavior of global leaders during the COVID-19 pandemic. In addition, we add experimental evidence on the impact of such role modelling from a low-income country context.

## 3 Presidents as vaccine ambassadors

We systematically collected information on the vaccine role modelling behavior of global leaders. We started from the October 2021 version of the Political Leaders’ Affiliation Database (PLAD), which contained information on the leaders of 173 countries around the world on December, 31, 2020 (37). If, by the time of the roll-out of the COVID vaccines in a country, the head of state had changed, we updated the PLAD dataset. Supplementary Appendix 1 describes our coding procedure in detail. Figure one graphically presents the data; Panel A indicates whether heads of state supported the vaccination campaign, Panel B indicates whether they got vaccinated themselves, Panel C indicates whether an image of the vaccination was made available to the public.

**Figure 1:**
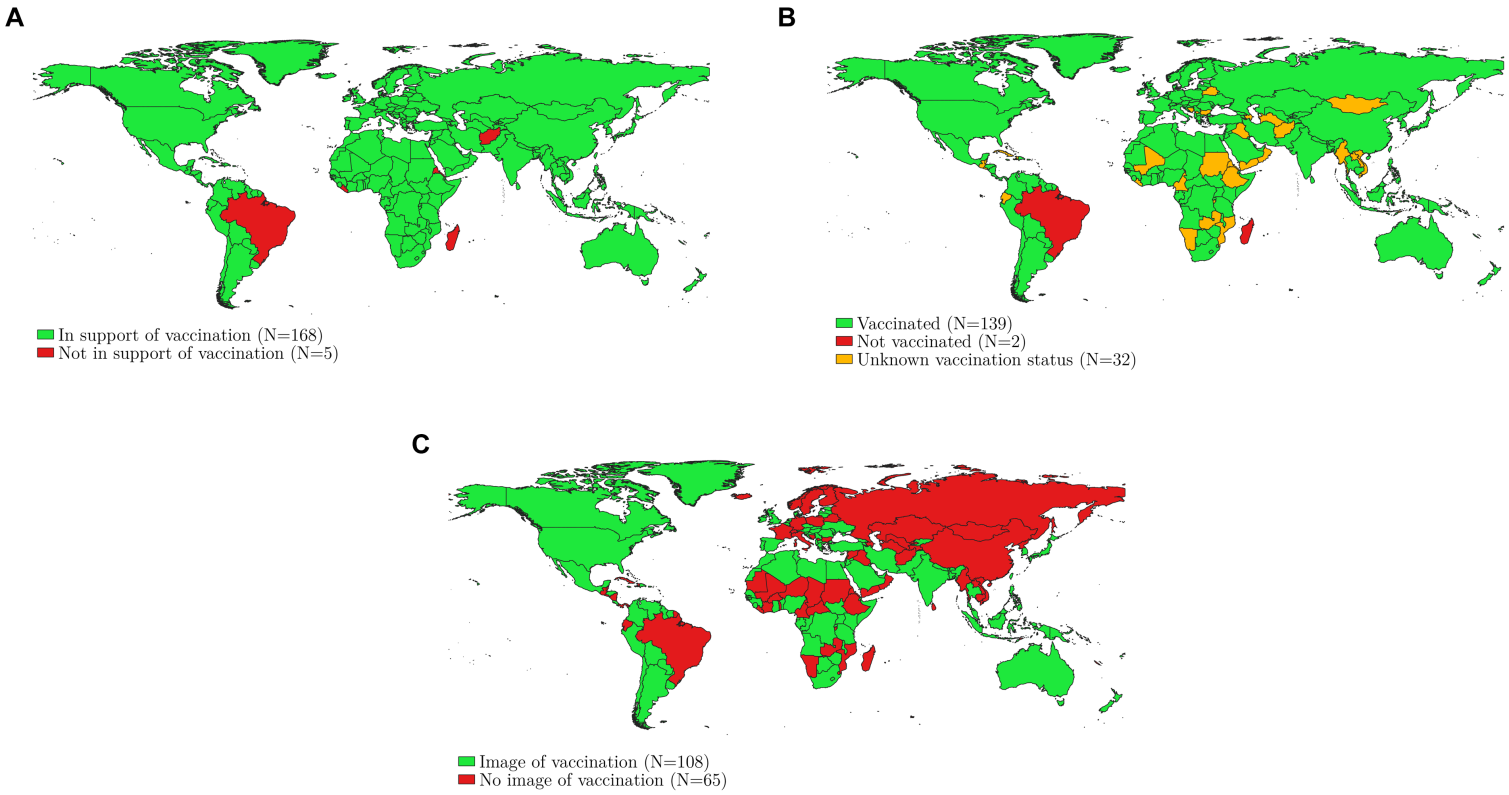
These maps provide information on 173 global leaders’ endorsement of COVID-19 vaccination. Panel **A** indicates whether heads of state supported the vaccination campaign, Panel **B** indicates whether heads of state got vaccinated themselves, Panel **C** indicates whether an image of the vaccination was made available to the public. Own compilation, Supplementary Appendix 1 provides coding details.

We find that 168 out of 173 leaders explicitly supported the vaccination campaign (Figure 1, Panel A). Among those who did not, are two very explicit anti-COVID-vaccine presidents – Madagascar’s Andry Rajoelina and Brazil’s Bolsonaro – and three presidents who ‘tolerated’ the vaccination campaign, despite a lack of personal acts or words to support it (President Isaias Afwerki from Eritrea, President George Weah of Liberia, and Supreme Leader of Afghanistan Hibatullah Akhundzada). We found that 139 leaders (80%) received a COVID-19 vaccine but could not confirm the vaccination status of 32 leaders (Figure 1, Panel B). Moreover, 108 leaders (78% of those vaccinated) distributed a picture or a video of their vaccination (Figure 1, Panel C). We can therefore conclude that most heads of state thought it was important to communicate their vaccination status to the public using more than words.

The extent to which heads of state can act as credible vaccine ambassadors likely depends on the public trust they enjoy. Systematic reviews documented that a lack of trust in governments is associated with vaccine hesitancy and refusal across a wide range of countries (38–40). While quantitative evidence for low-income countries is scant, existing studies point in the same direction. Blair et al. (41) and Vinck et al. (42), studying Ebola outbreaks in Liberia and DR Congo, find for instance that respondents with low trust in government institutions exhibit less compliance with recommended behavior changes and a lower willingness to take up an Ebola vaccine. Moreover, Stoop et al. (6), leveraging data from 22 African countries, highlight that institutional mistrust – including mistrust in the head of state – is an important barrier to reaching universal child immunization. Such trust is often context-dependent and varies across subsets of the population (43). In some groups, anti-establishment sentiment can be so high that vaccination support by certain public figures can backfire (28).

In what follows, we turn to the DR Congo to analyze the impact of President Tshesekedi’s COVID-19 vaccination on citizen’s vaccine acceptance.

## 4 COVID-19 in DR Congo

President Tshisekedi got vaccinated with Moderna on September 13^th^, 2021. This is late compared to other African presidents (44). He initially went against the vaccine promotion strategy of his own government by refusing to get vaccinated for 6 months, casting doubt on the AstraZeneca vaccine,^1^ and even promising to launch a Congolese ‘anti-COVID’ product at a meeting in Berlin in August 2021 (46). Less than 2 weeks after that statement, he made a U-turn and received his COVID-19 vaccine live on Congolese television. The news was distributed by diverse national media channels, Facebook, and Twitter.

It is not clear to what extent the news of Tshisekedi’s vaccination (and his initial reluctance) reached Congolese citizens. Since only 19.4% percent of households owns a television, only a minority would have been directly exposed to images of the president’s live vaccination. A larger proportion of households owns a radio (37.6%) or phone (51.8%). But, according to the latest data, internet penetration at home stands at only 1.3%, and a mere 1.5% of women and 5.5% of men aged 15–49 are estimated to access news on either radio, newspaper, or television on a weekly basis (13).

By the time President Tshisekedi got vaccinated, DR Congo had officially reported 56,000 COVID-19 cases and 1,066 deaths, or a death toll of just 0.0012% for a population of 90 million (44). But, with low testing and tracing capacity, these are likely underestimates. Looking at a highly visible (and exposed) subpopulation, namely members of parliament, the death toll reaches 5 % (47). Out of 640 Congolese parliamentarians, 32 died from COVID-19 (48). These high-profile cases fed the popular belief that COVID-19 is a disease of the urban elite, and therefore not a concern for ‘ordinary’ Congolese (49). Combined with conspiracy theories as well as the need of a largely poor population to provide in one’s livelihood, this led to overall low compliance with containment measures, such as lockdowns and restrictions on travel and public gatherings (50).

DRC received its first vaccines in March 2021, in the form of 1.7 million AstraZeneca doses from the COVAX vaccine sharing scheme, but rollout was delayed due to safety concerns, and eventually around 75 percent of these doses were re-exported to make sure they were used before they expired (48). September 2021 marked a new phase in the vaccination campaign, with the arrival of vaccines from Sinovac, Johnson & Johnson, Moderna and Pfizer (51–53). The president’s public vaccination with Moderna thus coincided with the arrival of mRNA vaccines in the country, and these were in first instance intended for 15 priority provinces, among which North-Kivu, where our research takes place (54). While vaccination rates increased with the arrival of these new vaccines (51), many Congolese remained reluctant. WHO statistics indicate that by December 2022 less than 7 doses per 100 population were administered in DRC (55).^2^ Only Yemen, Eritrea and Papua New Guinea ranked lower.

Aside from rumors and conspiracy theories, the low vaccination rate was compounded by the country’s limited healthcare infrastructure, low numbers of health workers and broader governance issues, including rampant corruption and political instability (56). These governance issues not only affect the country’s ability to provide basic services to its citizens but also erode general trust of Congolese citizens in public institutions and President Félix Tshisekedi. A December 2021 opinion poll by the Congo Research Group (57) revealed that only 29% of Congolese surveyed had a positive opinion of Tshisekedi. In contrast, the Congolese Cardinal, Fridolin Ambongo Besungu, was trusted by 47%.

Within this context, we analyze the potential of president Tshisekedi to act as a vaccine ambassador and influence Congolese citizens’ COVID-19 vaccine acceptance by getting publicly vaccinated himself.

## 5 Data and methods

### 5.1 Data collection

**Figure 2:**
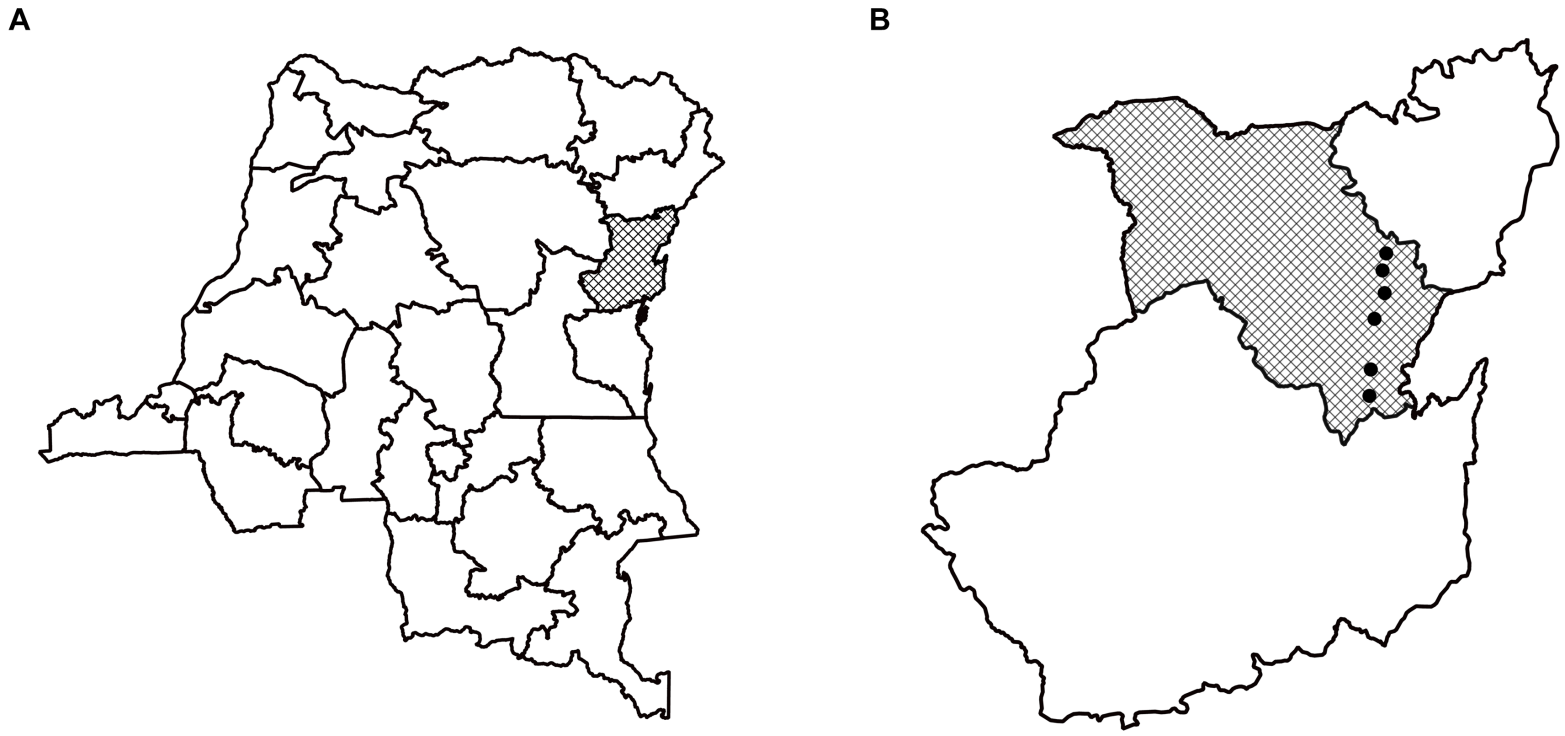
Panel **A** indicates the province of North-Kivu within the DRC. Panel **B** indicates the territory of Lubero within North-Kivu and the location of our six study villages.

We present results based on 600 in-person interviews conducted in the period September–October 2021. Our survey took place in Lubero territory, one of the six territories that make up North Kivu, a province that spans almost 60,000 km^2^ (Figure 2). The province contains abundant natural resources, encompassing minerals, biodiverse protected areas, and fertile agricultural land. It has however been plagued by violence for over two decades, and currently still counts more than 100 armed groups within its borders (58). Lubero territory is predominantly rural, and the majority of residents engage in agriculture.

Within Lubero territory, we conducted surveys in six localities (Figure 2, Panel B). These localities were selected because they were part of a broader ongoing study on the impact of electricity provision in communities nearby Virunga national park (59). A team of 16 enumerators first conduced a census in each locality, yielding a total of 11,577 observations on households’ geographic position and their socio-economic status through visual checks of the house and its construction materials. We then randomly selected 600 households to be surveyed, stratified by the construction quality of their houses, and proportional to the population size of the locality.

### 5.2 Measuring vaccine acceptance and institutional trust

The survey recorded respondent’s willingness to get vaccinated through the question “Let us assume a vaccine against Coronavirus was available for you, would you take it?.”^3^ Answers were given on a four-point Likert scale (certainly, probably, probably not, certainly not). Our binary measure for stated vaccine acceptance equals one for those respondents who indicated they would certainly or probably take a COVID-19 vaccine, and zero otherwise.

Our survey also measured institutional trust. Building on an earlier study in the same region (42) we asked the following question, for five institutional levels (local, municipality, provincial, national, president) and the cardinal: “In general, to what extent do you believe the following authorities represent the best interests of the Congolese population?.” We then repeated this question specifically about these actors’ management of the COVID-19 crisis. Respondents were asked to indicate their trust on a scale from 1 to 5 (1 being associated with the highest level of trust, and 3 being neutral). We recategorized those variables as binary taking the value one if a respondent indicated a trust value of 1 or 2, and zero otherwise.

### 5.3 Design

We embedded a randomized survey experiment in the questionnaire. Before answering the question on vaccine acceptance, 1/3 of respondents (*N* = 203) was prompted to consider the hypothetical vaccination of their president: “Assume the president, Félix Tshisekedi, were to take the vaccine live on television.” As Congolese have little trust in the president, but relatively high trust in the church, another 1/3 of respondents (*N* = 202) was prompted to consider the hypothetical vaccination of their cardinal: “Assume the cardinal, Fridolin Ambongo Besungo, were to take the vaccine live on television.” The remaining 1/3 of respondents (*N* = 195) was directly asked the question on vaccine acceptance, without a prompt. We compare the stated vaccine acceptance of the respondents in the two treatment groups to that of the control group.

To our own surprise, President Tshisekedi got publicly vaccinated while our survey was ongoing, and we had already interviewed 114 (19%) of our respondents. We take advantage of this opportunity to analyze whether stated vaccine acceptance differed between respondents surveyed before and after Tshisekedi’s public vaccination. However, since Lubero territory is remote and poorly endowed with public infrastructure, many respondents have no direct access to news outlets, thus were likely not aware of the presidential vaccination. We explore the role of information transmission by leveraging the following question, which we added in our surveys conducted after the president’s public vaccination: “Do you think the president, Félix Tshisekedi, received a vaccine against Coronavirus?”

### 5.4 Statistical analyses

The survey experiment relies on a randomized design, resulting in treatment and control groups that are balanced on average across all observed covariates (see Supplementary Appendix 2). We can hence investigate its impact by comparing mean stated vaccine acceptance across those treated and untreated relying on t-tests. In addition, we run a set of multivariable logistic regressions with stated vaccine acceptance as the outcome variable. Specifically, we estimate the following specification:

(1) $$\mathrm{prob}\;\left(Y_{i}=1\right)=\Phi \left(\beta P_{i}+\gamma T_{i}+X_{i}^{\prime }\theta \right)$$

where $Y_{i}$ is respondent *i*’s stated vaccine acceptance. The variable P indicates whether respondent *i* received the prompt about the hypothetical vaccination of the president or the cardinal. Coefficient *β* is estimated from the model and represents the treatment effect. Variable T measures a respondent’s trust in the president or cardinal with respect to COVID-19. Vector X includes a range of control variables that may correlate with vaccine acceptance. At the level of the respondent, we include age, gender, years of education and ethnicity. We further control for respondents’ stated opinions regarding the importance, effectiveness, and safety of vaccines as well as their compatibility with respondents’ religious beliefs. At the household-level, we control for household size, dependency ratio, log yearly income, construction quality of the home, and the ownership of radio and television.

To analyze the moderating role of trust in the survey experiment, we add an interaction term to equation (1):

(2) $$\mathrm{prob}\;\left(Y_{i}=1\right)=\Phi \left(\beta P_{i}+\gamma T_{i}+\delta P*T_{i}+X_{i}^{\prime }\theta \right)$$

where variable T measures a respondent’s trust in the president or cardinal with respect to COVID-19, and coefficient δ allows to investigate how the effect of the survey experiment differed for respondents with high or low trust.^4^

To investigate the impact of the president’s actual vaccination on stated vaccine acceptance, we estimate:

(3) $$\mathrm{prob}\;\left(Y_{i}=1\right)=\Phi \left(\beta P_{i}+\gamma T_{i}+\lambda V_{i}+\nu A_{i}+X_{i}^{\prime }\theta \right)$$

where the variable V indicates whether a respondent was interviewed after Tshisekedi’s public vaccination. Here we are interested in exploring the role of information transmission. This is captured by variable A, indicating whether a respondent was aware of the president’s vaccination. Such awareness may be correlated with other characteristics that can influence vaccine acceptance, e.g., perhaps it captures respondents who are more informed in general, and therefore also about health benefits of vaccination. To control for such possible confounding factors, we augment vector X with variables capturing respondents’ knowledge of politics and include measures to capture how often they listened to the radio or watched television in the week prior to the interview.

Although specification (3) controls for a large range of potentially confounding variables, it remains possible that other unobserved characteristics simultaneously influence awareness of the president’s vaccination and vaccine acceptance. To formally assess the threat of such omitted variable bias, we turn to the procedures suggested by Altonji et al. (60) and Oster (61). It uses selection on observable variables as a guide to assess the potential bias from unobserved variables. Selection on observable variables is evaluated by looking at movements in the estimated coefficients on the awareness variable while gradually controlling for additional covariates. The relevance of these covariates is assessed by evaluating associated movements in the R-squared. Based on these insights, Oster developed a measure that allows to assess how large selection on unobservable variables has to be, relative to selection on observables, to fully explain away the estimated effect (Supplementary Appendix 4 describes the methodology in detail).

## 6 Results

### 6.1 Descriptive statistics

**Figure 3:**
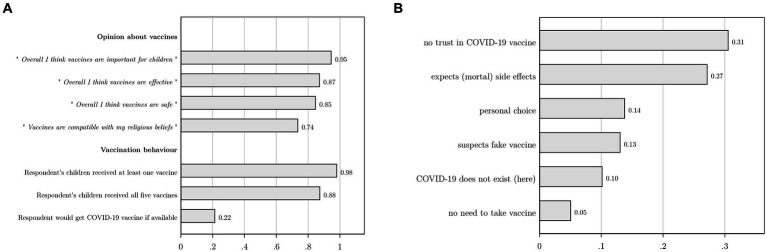
We asked respondents with children (97.5%) whether their children were vaccinated against tuberculosis, diphtheria, polio, measles, and yellow fever. In Panel **A**, the first two bars under the heading ‘Vaccination behavior’ indicate whether children received at least one of these vaccines, or all five vaccines. Panel **B** represents the answer categories that emerged after categorizing open answers to the question “Why would you not take a COVID-19 vaccine?.” This question was asked to the 412 respondents who indicated not to accept a COVID-19 vaccine if it would be available to them; 129 respondents indicated they would accept it, while the remaining 59 respondents refused to answer or indicated they were unsure.

On average, a sample respondent is 44 years old and has 6.8 years of education (Table 1). About one third of respondents are male. The average household counts 6.6 members, with a dependency ratio of 0.53, indicating that about half of members are not in the active age group (15 to 60). The mean annual household income is 947 USD, corresponding to a local purchasing power of 1,894 USD in 2021, thus implying 5.2 USD PPP per day. Almost half of households own a radio, while only 13% owns a television. Most respondents agree that vaccines are important for children (95%), effective (87%), safe (85%) and compatible with their religious beliefs (74%). Consequently, general vaccine acceptance is very high (Figure 3, Panel A). Almost nine out of ten households indicate to have vaccinated their children against tuberculosis, diphtheria, polio, measles, and yellow fever, while 98% of households vaccinated their children against at least one of these diseases. In sharp contrast, only 22% of respondents indicated they would accept the COVID-19 vaccine if it was available to them. This is much lower than the mean stated COVID-19 acceptance rate of 80% found in a sample of 10 low- and middle-income countries in Asia, Africa and South America, but in line with other studies reporting lower COVID-19 vaccine acceptance and confidence in African countries and the DR Congo in particular (2, 9, 62).

**Table 1:** Socio-demographic profile of respondent and household.

	Obs.	Mean	Std. dev.	Min	Max
Respondent’s age	600	44	16	18	79
Respondent is male	600	0.31	0.46	0	1
Respondent’s years of education	600	6.82	4.41	1	18
Respondent is of dominant ethnicity	600	0.99	0.11	0	1
Household size	600	6.59	2.72	1	22
Household dependency ratio	600	0.53	0.20	0	1
Household yearly income ($)	600	947	1,490	0	9,250
Household owns radio	600	0.49	0.50	0	1
Household owns television	600	0.13	0.33	0	1

The dependency ratio is calculated as the number of people younger than 15 plus the number of people older than 64 divided by the total size of the household.

Respondents with a stated willingness to take the vaccine (*N* = 129) indicated they would do so to protect themselves (91%), their family and household (78%) and their community (63%). Respondents who did not accept the vaccine (*N* = 412) were asked to motivate their choice in an open question. After categorizing their open answers, six main answer categories emerged (Figure 3, Panel B). The largest group among them (31%) indicated a general lack of trust in the COVID-19 vaccine and its efficacy. About 27% expected that they might get COVID-19 from the vaccine or feared other, potentially mortal, side-effects. Illustrative answers included “To avoid Corona contamination,” “This vaccine kills people” and “It’s poison.” More than 1 out of 10 (13%) suspected that they would not receive a real vaccine, mentioning, e.g., “It’s a fake vaccine,” “It’s a bad vaccine. White people want to eliminate us,” “The vaccine sent to Africa is dubious.” Others indicated that it is their personal choice not to take the vaccine (14%), that they doubted the existence of COVID-19 (10%), or they felt no need to take the vaccine as they believed they would not get sick (5%).

**Figure 4:**
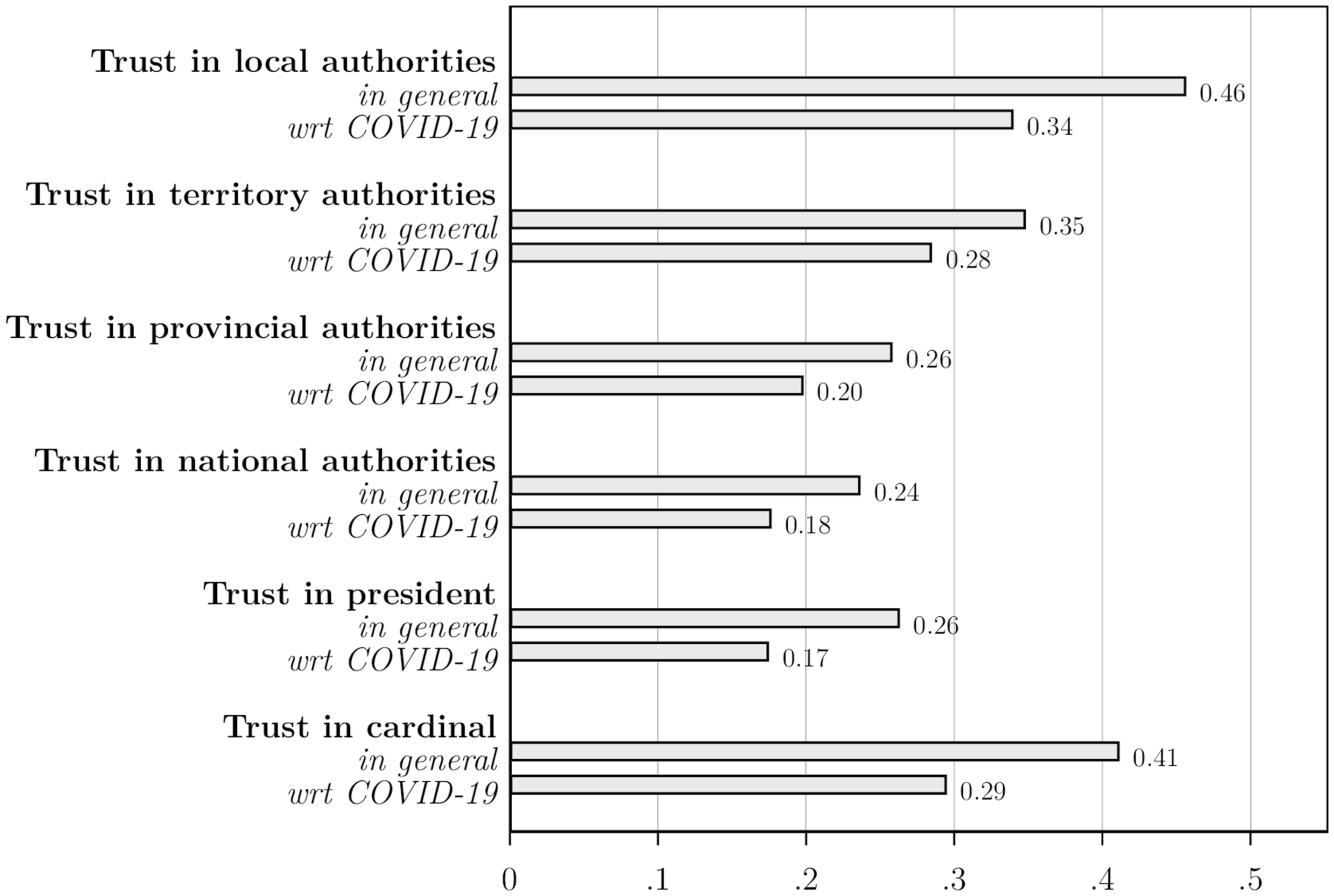
For each institutional level, we asked: “In general, to what extent do you believe the following authorities represent the best interests of the Congolese population?.” We then asked this question specifically related to these authorities’ management of the COVID-19 crisis. *N* = 600.

We find rather low levels of institutional trust, ranging between a low of 17% for the president with respect to his management of the COVID-19 crisis and a high of 46% for general trust in local authorities (Figure 4). Overall, we find that institutional trust is systematically lower within the COVID-19 context, and systematically lower for institutions higher up in the administration. In line with the opinion poll by the Congo Research Group (57), trust in the cardinal is considerably higher than trust in the president.

### 6.2 Survey experiment

**Figure 5:**
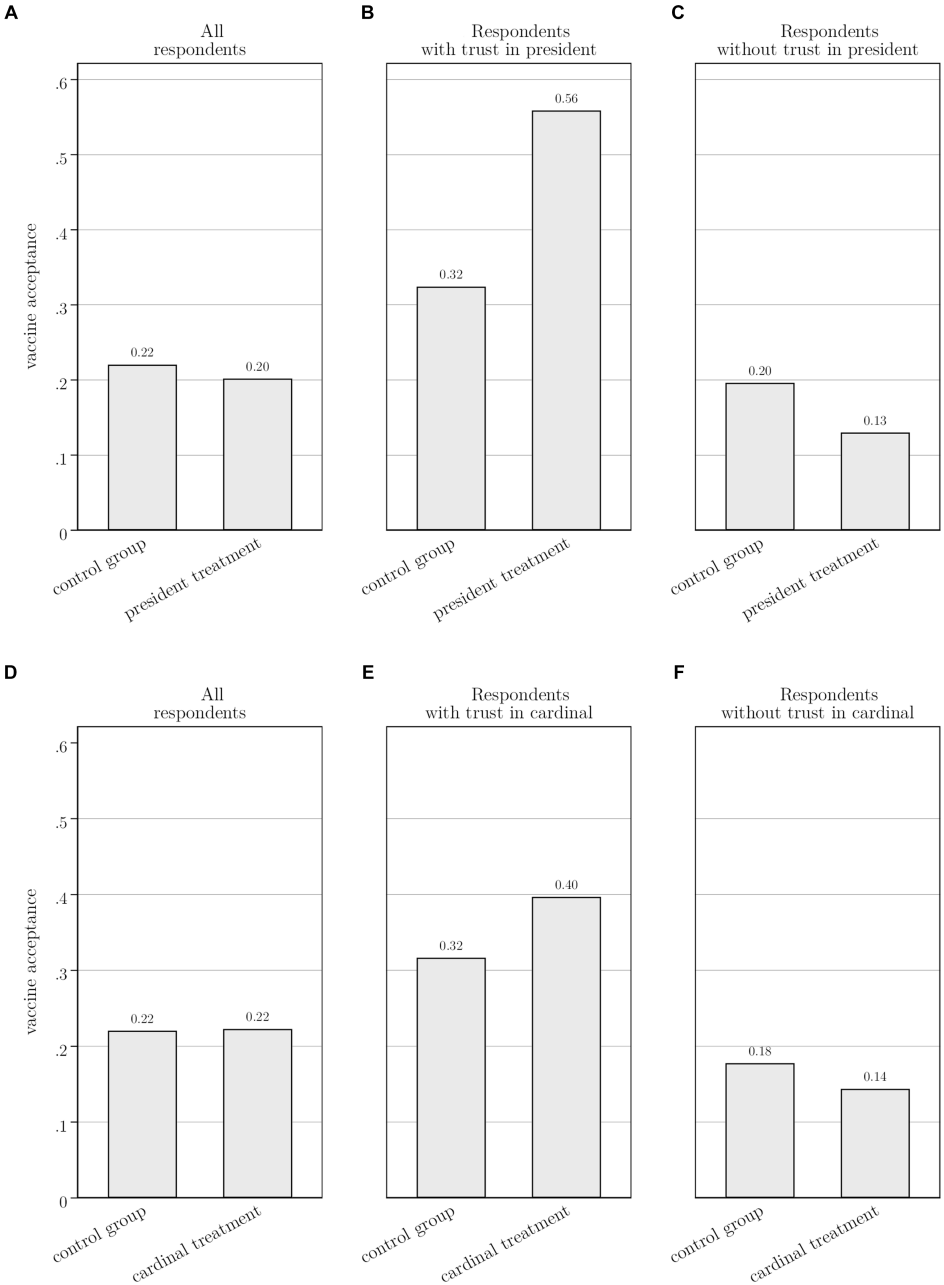
Panel **A** compares vaccine acceptance across respondents in the president treatment (*N* = 203) and the control group (*N* = 195). The difference in means is 0.02 (*p*-value:0.65). Panel **B** only considers respondents who trust the president with respect to COVID-19 (*N* = 71). It compares vaccine acceptance across respondents in the president treatment (*N* = 37) and the control group (*N* = 34). The difference in means is 0.23 (p-value:0.047). Panel **C** only considers respondents who do not trust the president with respect to COVID-19 (*N* = 327). It compares vaccine acceptance across respondents in the president treatment (*N* = 158) and the control group (*N* = 169). The difference in means is 0.07 (p-value:0.11). Average trust in the president is balanced across the control (0.19) and treatment (0.17) group, with a difference in means of 0.02 (*p*-value:0.56). Panel **D** compares vaccine acceptance across respondents in the cardinal treatment (*N* = 202) and the control group (*N* = 195). The difference in means is 0.002 (*p*-value:0.96). Panel **E** only considers respondents who trust the cardinal with respect to COVID-19 (*N* = 123). It compares vaccine acceptance across respondents in the cardinal treatment (*N* = 63) and the control group (*N* = 60). The difference in means is 0.08 (p-value:0.36). Panel **F** only considers respondents who do not trust the cardinal with respect to COVID-19 (*N* = 274). It compares vaccine acceptance across respondents in the cardinal treatment (*N* = 139) and the control group (*N* = 135). The difference in means is 0.03 (*p*-value:0.45). Average trust in the cardinal is balanced across the control (0.31) and treatment (0.31) groups, with a difference in means of 0.004 (*p*-value:0.93). Differences in means and significance levels are obtained from *t*-tests.

Figure 5 presents the results of the survey experiment, relying on t-tests to assess differences in means between the control group and the treatment group.^5^ On average, the hypothetical vaccination of President Tshisekedi has no effect on vaccine acceptance (Panel A). However, we find trust in the president to be an important moderating variable. Among respondents who trust the president, exposure to his hypothetical vaccination raises vaccine acceptance with 24 percentage points, from 0.32 to 0.56, a sizeable difference that is significant at the 5%-level (Panel B). Among respondents who indicated not to trust the president, vaccine acceptance is seven percentage-points lower among those in the treatment group (0.13 compared to 0.20), but, with a *p*-value of 0.11, the result is just shy of being statistically significant at the 10%-level (Panel C).

The hypothetical vaccination of the cardinal in the survey experiment failed to boost vaccine acceptance, even among respondents who indicated to trust the cardinal (Panels D–F). Despite the higher perceived trustworthiness of the cardinal, these results suggest that the Cardinal’s actions play no role in influencing respondents’ vaccine acceptance. This aligns with the conclusion of Abu-Akel et al. (29) that, in times of health crises, it is health experts and government officials – those in a position to manage the crisis and be held accountable for it – who are likely to exert the greatest influence on public opinion.

These findings are confirmed in a multivariable logistic regression that controls for the respondent- and household level covariates identified above (Table 2). The results in Column (1) relate to equation (1). We find that, on average, neither the president treatment nor the cardinal treatment in the survey experiment significantly affected vaccine acceptance. Our results do confirm the importance of institutional trust; respondents who trust the president when it comes to managing the COVID-19 crisis are twice more likely to indicate that they are willing to get a COVID-19 vaccine (*p* < 0.01). In contrast, trust in the cardinal is not associated with stated vaccine acceptance. In Column 2, we estimate equation (2) and include interaction terms to explore how trust in the president and the cardinal affect the survey experiment treatment effects. We find that public trust strongly reinforces the impact of the president treatment. Specifically, stated vaccine acceptance for respondents who trust the president and were exposed to the president treatment is 4.75 times higher (*p* < 0.01) than that of respondents in the base category (those who do not trust the president and were not exposed to president treatment).^6^ The results do not indicate a statistically significant interaction between the cardinal treatment and trust in the cardinal.

**Table 2:** Multivariable logistic regressions.

	**Willingness to get COVID-19 vaccine**				
	(1)	(2)	(3)	(4)	(5)
	OR [95% CI]	OR [95% CI]	OR [95% CI]	OR [95% CI]	OR [95% CI]
President treatment	0.93	0.60^*^	0.93	0.91	0.59^**^
	[0.50,1.74]	[0.36,1.00]	[0.50,1.72]	[0.50,1.67]	[0.35,0.98]
Cardinal treatment	1.06	0.74	1.05	1.04	0.77
	[0.68,1.63]	[0.38,1.44]	[0.69,1.61]	[0.68,1.60]	[0.41,1.45]
Trust in president WRT COVID	3.01^***^	1.80	3.00^***^	3.00^***^	1.77
	[1.61,5.62]	[0.71,4.55]	[1.60,5.65]	[1.60,5.62]	[0.68,4.57]
Trust in cardinal WRT COVID	1.71	1.33	1.72	1.63	1.35
	[0.80,3.67]	[0.68,2.61]	[0.81,3.66]	[0.67,3.97]	[0.65,2.79]
President treatment * trust president WRT COVID		4.75^***^			5.04^**^
		[1.64,13.81]			[1.45,17.52]
Cardinal treatment * trust cardinal WRT Covid		2.31			1.97
		[0.80,6.67]			[0.66,5.87]
Interviewed after president’s vaccination			1.14	0.88	0.93
			[0.56,2.29]	[0.43,1.78]	[0.45,1.92]
Aware that president got vaccinated				2.97^***^	2.95^***^
				[1.74,5.06]	[1.70,5.14]
Socio-demographic controls	Yes	Yes	Yes	Yes	Yes
Vaccine confidence indicators	Yes	Yes	Yes	Yes	Yes
Knowledge of politics	No	No	No	Yes	Yes
Radio & television usage	No	No	No	Yes	Yes
Observations	600	600	600	600	600
Pseudo *R*^2^	0.12	0.14	0.12	0.14	0.16

Data are Odds Ratios from a logistic regression with respondent’s willingness to get a COVID-19 vaccine as the outcome variable. Standard errors are clustered at the village-level. Significance is indicated by *** *p* < 0.01, ** *p* < 0.05, * p < 0.10. Full regression output is presented in Supplementary Appendix 3.

### 6.3 Impact of the president’s actual vaccination

**Figure 6:**
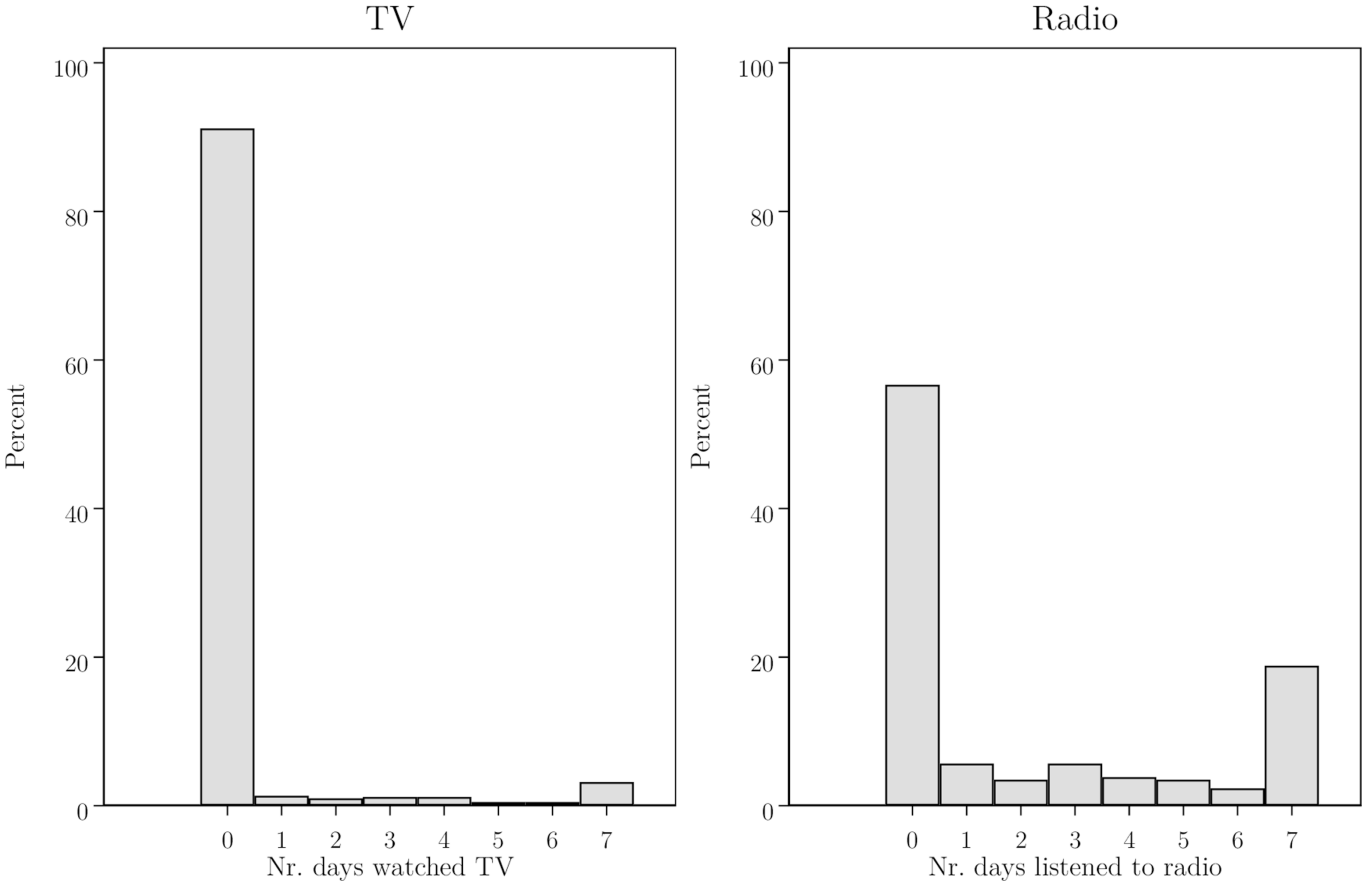
We asked respondents to indicate the number of days that they watched TV and listened to the radio in the week prior to the interview. This Figure presents histograms based on the full sample of respondents (*N* = 600).

Four out of five respondents (486 out of 600) were interviewed *after* the broadcasting of President Felix Tshisekedi’s vaccination. However, media access is low in our study area, and the news may not have reached everyone. For instance, Figure 6 shows that the large majority of respondents did not watch television (91%) or listen to the radio (57%) in the week prior to the interview. Hence, it is no surprise that only 89 respondents reported being aware of the President’s inoculation.^7^ The actual exposure to the president’s vaccination is thus much smaller, covering just 18% of the sample interviewed after the president got vaccinated.

In Column 3 of Table 2 we add an indicator variable for respondents who were interviewed after President Tshisekedi got vaccinated on 13 September 2021. In Column 4, we further add a variable that captures whether a respondent was aware of the president’s vaccination. In addition, we add variables capturing respondents’ knowledge of politics and include measures to capture how often they listened to the radio or watched television in the week prior to the interview – thereby estimating equation (3). The results show that being interviewed after the president’s vaccination does not by itself affect stated vaccine acceptance. We only find an impact for those who indicated being aware of the president’s vaccination; these respondents are 197% more likely to indicate that they are willing to get a COVID-19 vaccine (*p* < 0.01).^8^

Being aware of the president’s vaccination may be correlated with other characteristics that can influence vaccine acceptance. While we control for a large set of likely confounding covariates, it is possible that other, unobserved, characteristics are driving our findings. Relying on the procedures suggested by Altonji et al. (60) and Oster (61) we formally assess the threat of such omitted variable bias. We find that selection on unobservables would have to be 5.97 times larger than selection on the included variables to fully explain away our estimated effects on awareness of the president’s vaccination. Appendix 4 discusses the methodology and results in detail. Taken together, the findings suggest that our qualitative conclusions are not sensitive to omitted variable bias.

### 6.4 The impact of public trust and media outreach

**Figure 7:**
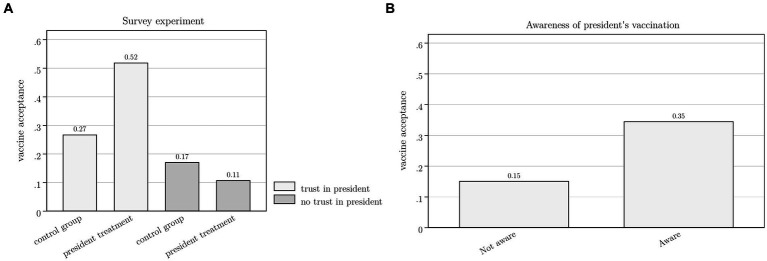
This Figure is based on the multivariable logistic regression presented in column 5 of Table 2. Panel A presents predictive margins implying that for Congolese who report trusting the president, the president treatment in the survey experiment boosted vaccine acceptance from 0.27 (95%-CI: 0.18 to 0.35) to 0.52 (95%-CI: 0.21 to 0.83). For those who mistrust the president, it decreased acceptance from 0.17 (95%-CI: 0.08 to 0.27) to 0.11 (95%-CI: 0.02 to 0.19). All other covariates are held at their mean values. The predictive margins in Panel B implies that being aware of the president’s vaccination, while holding all other covariates at their mean values, increases vaccine acceptance from 0.15 (95%-CI, 0.08 to 0.23) to 0.35 (95%-CI, 0.14 to 0.55).

Our results demonstrate that the impact of the president’s vaccine role modelling is moderated by trust and depends on media outreach and access. In Figure 7 we make our findings more concrete by presenting predictive margins based on the most inclusive regression specification presented in Table 2. Panel A presents predictive margins for the survey experiment, by trust in the president. Holding all other covariates at their mean values, we find that for Congolese who report trusting the president, the experiment strongly boosted vaccine acceptance from 27 to 52%, nearly a doubling. However, for those who mistrust the president, the survey experiment decreased acceptance from 17 to 11%. Panel B focuses on the president’s public vaccination. The estimated predictive margins imply that being aware of the president’s vaccination, while holding all other covariates at their mean values, increases vaccine acceptance from 15 to 35%.

## 7 Conclusion

When systematically documenting the attitudes and behavior of heads of state regarding COVID-19 vaccination, the picture that emerges is overwhelmingly pro-vaccine: almost all global leaders endorsed the vaccination rollout, 80% publicly announced their vaccination and 62% did so with a picture or a video. We can thus conclude that most heads of state thought it was important to communicate their vaccination status to the public using more than words.

The cost of a leader getting vaccinated publicly is very low, but its symbolic value may be high, as demonstrated in recent studies from the United States. It is however unclear to what extent these results travel to different settings. In our DR Congo case study, we relied on a survey experiment to empirically verify the impact of such vaccine role modelling. The results indicate that the president’s hypothetical inoculation only increases vaccine acceptance among those who trust the president, while it depresses vaccine acceptance among those who do not. When the president got publicly vaccinated during the survey period, we find that it only increased vaccine acceptance among respondents who were aware of this fact.

These results have important policy relevance. They show that public vaccination of heads of state can only effectively serve as a vaccination advocacy tool if two conditions are satisfied. First, the said leader should be perceived as trustworthy by citizens. Second, the live inoculation should be widely communicated, preferably through diverse channels that also reach areas with low media access. These conditions were largely absent in our study area. Only 17% of respondents expressed trust in the president amidst the COVID-19 crisis, and only 18% of those interviewed after the president got vaccinated were aware of his vaccination. In such a context, vaccination of local public figures, for instance village leaders or respected older adult community members, might be more effective to improve vaccine acceptance. Indeed, our data shows that trust in local leaders is almost twice as high as trust in the president. Additionally, despite the remoteness of these territories, local news travels through word of mouth, as communities are tightly knit and easily exchange information. Mind however that our null result regarding the cardinal’s hypothetical vaccination suggests that it is not sufficient to pick any well-known and well-trusted person. Future research should delve deeper into identifying suitable ‘vaccine ambassadors’ across varied contexts.

## Data availability statement

The data and replication files for the analyses presented in this article are publicly available at https://doi.org/10.7910/DVN/OAYJCV.

## Ethics statement

The studies involving humans were approved by the University of Antwerp Ethical Advice Committee (file SHW_19_03). The studies were conducted in accordance with the local legislation and institutional requirements. The ethics committee/institutional review board waived the requirement of written informed consent for participation from the participants or the participants’ legal guardians/next of kin because we anticipated that many of our participants would be illiterate. In addition, given the local context, asking them to sign a document may even create suspicion. Hence, we provided participants with a verbal description of the study and its purpose. Verbal consent was sought from the participants and they were informed of their right not to participate or not to answer certain questions. Participants further had the option to discontinue the survey at any given point without consequences.

## Author contributions

LC: Formal analysis, Writing – original draft, Writing – review & editing. EL: Conceptualization, Formal analysis, Investigation, Methodology, Writing – original draft, Writing – review & editing. NS: Conceptualization, Formal analysis, Investigation, Methodology, Writing – original draft, Writing – review & editing. MV: Conceptualization, Formal analysis, Investigation, Methodology, Writing – original draft, Writing – review & editing.

## Footnotes

^1^In late 2020, AstraZeneca paused the rollout of its vaccine after reports emerged of a small number of people who had developed blood clots after receiving the vaccine. This led to some concerns among the public about the safety of the vaccine and prompted some governments to temporarily suspend its use (45).

^2^By March 2022, 5.7% of the population received at least one dose of a vaccine and 1.03% were fully vaccinated. Of those vaccinated, 48% received the Johnson & Johnson vaccine, while 42% received an mRNA vaccine (25% Moderna and 17% Pfizer), 7% received Sinovac and 2% AstraZenica (51).

^3^At the time of the survey, COVID-19 vaccines were not yet available in the study region.

^4^Results for specification (1) and (2) remain qualitatively unchanged when additionally controlling for (awareness of) the president’s vaccination through variables V and A (see Table 2).

^5^Table A2 in Supplementary Appendix 2 shows that covariates are balanced between the control and treatment group.

^6^To help interpret this finding it is useful to explore predictive margins, which we do in Figure 7.

^7^Watching television and listening to the radio are positively and significantly correlated with being aware of the President’s vaccination and general knowledge about politics (measured through a question asking them to name the president of Uganda). Correlation coefficients range between 0.16 and 0.28 and are all significant at the 1%-level. In addition, 13% of our respondents indicated that their household does not own a mobile phone, and among those that do own one, only half of respondents reported to use it on a daily basis. While we cannot infer from out data whether respondents have access to the internet, national statistics indicate that only 3.6% of women and 11.3% of men aged 15–49 use the internet at least once per week [INS, USAID, and UNICEF (13)]. We did ask respondents to what extent they trusted social media as a source of news on COVID-19. Answer categories included (1) high trust, (2) little trust, (3) no trust at all. Only 5% indicated high trust, while 37% indicated little trust and the majority (59%) indicated not to trust social media at all as a source of news on COVID-19.

^8^It is possible that being aware of the president’s public vaccination influenced the results of the survey experiment. We check this in Column 5 and find that the results are qualitatively unchanged compared to Column 2. We further explored whether there is a significant interaction between trust in the president and being aware of his vaccination. The results indicate that this is not the case, but this may be due to power issues, given that only 3.3% of the sample indicates to trust the president and be aware of his vaccination.
