# Supplementary material for: Presidents and vaccines: head of state inoculation as a tool for vaccine promotion
Source: Front Public Health. 2024 May 14;12:1364927. doi: 10.3389/fpubh.2024.1364927 (PMC11131420; doi:10.3389/fpubh.2024.1364927)
Supplement: Supplementary file 1 [file Data_Sheet_1.pdf]

Presidents and Vaccines

Head of state inoculation as a tool for vaccine promotion

Supplementary Material

Contents

1 Global leaders' COVID-19 vaccine role modelling 2

2 Survey experiment – covariate balance 4

3 Full regression output 5

4 Assessing Omitted Variable Bias 6

References 8

1 Global leaders' COVID-19 vaccine role modelling

We created three variables that document the attitudes and behavior of heads of states towards COVID-19 vaccines:

- **'image'**: 'yes' if we were able to find a photo or video of the head of state taking a COVID-19 vaccine, 'no' otherwise
- **'vaccinated'**: 'yes' if we were able to find information that the head of state took a COVID-19 vaccine, 'no' if we found information that the head of state refused to take a COVID-19 vaccine, and 'unknown' otherwise.
- **'support'**: 'yes' if we found information that indicated that the head of state supported the COVID-19 campaign, 'no' if we found evidence for the contrary. For this variable there were no 'unknown' as we could determine in all cases whether or not the head of state supported the campaign.

To determine who was the effective head of state at the time of vaccine roll-out, we started from the October 2021 version of the Political Leaders' Affiliation Database (PLAD), which contained information on the effective leaders of 173 countries around the world on December, 31, 2020 (Dreher et al. 2021). It follows the approach of the earlier Archigos database on Political Leaders which labels 'the person that de facto exercised power' as the effective leader (Goemans, Gleditsch, and Chiozza 2009).

The first COVID vaccines were administered in December 2020, but the first vaccination wave was mainly rolled out in the course of 2021. Hence, we verified if, compared to the PLAD December 2020 situation, the head-of-state had changed in the course of 2021. We did so through an internet search, relying on the list of Heads of State from the UN's Protocol and Liaison Service (UN, 2022), and a comparison of Wikipedia's 2020 and 2022 version of 'List of current heads of state'. If we found a change in the head-of-state by the time of the actual roll-out of the COVID-vaccines, we updated the PLAD database. In total, we replaced 14 heads of state.

To provide values for the three variables listed above, we conducted an internet search. We googled the name of each head of state in combination with the name of the country and the word 'vaccinated'. For instance, "Emmanuel Macron, France, vaccinated". If we found a picture or video of Macron getting a COVID vaccine, we set all three variables to 'yes'. If we did not find a picture, we repeated the search in the country's official language. If this search also did not result in an image, we set the variable 'image' to 'no', and then moved to the variable 'vaccinated', searching for news items, Facebook posts or tweets that revealed the leader's vaccination status (using the same search combinations). If we found that – despite the lack of an image – the leader was vaccinated, we set the variables 'vaccinated' and 'support' to 'yes' (which was the outcome in the case of Emmanuel Macron). If we found no information on the leader's vaccination status, we set 'vaccinated' to 'unknown'. If we found information that indicated that the leader refused to receive a COVID vaccine, we set 'vaccinated' to 'no'. We then searched for information on whether the leader supported the vaccination campaign. We set 'support' to 'yes' if we found news items that indicated active support by the leader, for instance the leader calling on the population to get vaccinated or attending an official gathering to receive a shipment of vaccines.

To verify the reliability of this procedure, we triangulated our results with those from a systematic overview of the vaccination status of African heads of state, as published in the September 2021 edition

of *Jeune Afrique* (n°3104). Comparing the information from our search with that of *Jeune Afrique* for the 52 African heads of state in our database, we found that in three cases where we had indicated 'unknown' as vaccination status, *Jeune Afrique* did have sufficient indications to report that the head of state was vaccinated. In five cases, we had confirmed knowledge on the leader's vaccination status while *Jeune Afrique* reported 'unknown' or 'no', which probably related to the fact that more information emerged in the time gap between the *Jeune Afrique* September 2021 issue and our writing. We updated our dataset accordingly. This comparison implies a margin of error of 5.8%, which is the grain of salt with which our data on leaders' vaccination status should consequentially be taken.

Finally, we set out to explore the correlation between global leaders' COVID-19 vaccine role modelling and country-level COVID-19 vaccination rates. We obtained information on country-level vaccination rates from a global database that is described in Mathieu et al. (2021) and freely available through Our World in Data. We extracted information on two variables: 1) the share of people who received at least one dose of a COVID-19 vaccine, and 2) the share of people who received all doses prescribed by the initial COVID-19 vaccination protocol. The database provides information on the evolution of these variables over time – we extracted the latest available information, hence indicating the highest recorded share for each country. We were able to obtain this information for 98% of the 173 countries in our database. For Eritrea, Eswatini and North-Korea there was no information available on either variable, while for Switzerland there was no information on the % of people who received all doses prescribed by the initial COVID-19 vaccination protocol. Besides the main 173 countries in our database, we have information on 55 overseas departments and islands such as Guadeloupe (France), Curaçao (Netherlands) and Greenland (Denmark). For 55% of these areas, we were able to extract information on COVID-19 vaccination rates.

In Table A1 we compare average COVID-19 vaccination rates across countries whose leaders displayed varying levels of vaccine role-modelling behavior. We find that vaccination rates are significantly higher in countries where the leaders got vaccinated and publicly shared an image of their vaccination. While this is in line with our expectation, we cannot infer causality from this observational analysis. Hence, we turn to a case study in the DR Congo where we can control for unobserved confounding factors through a randomized survey experiment and multivariable logistic regressions.

Table A1. Leaders' role modelling & COVID-19 vaccination rates

% of people who received at least one dose of a COVID-19 vaccine

	Yes		No		T-test	
	N	mean	N	mean	diff	p-value
Leader in support of vaccination	191	61.84	4	54.20	7.64	0.54
Leader vaccinated	162	64.09	33	49.85	14.24	0.002
Leader shared image of vaccination	130	64.53	65	55.98	8.54	0.02

% of people who received all doses prescribed by the initial COVID-19 vaccination protocol

	Yes		No		T-test	
	N	mean	N	mean	diff	p-value
Leader in support of vaccination	190	56.78	4	51.28	5.50	0.66
Leader vaccinated	161	58.73	33	46.62	12.11	0.01
Leader shared image of vaccination	130	58.93	64	52.07	6.86	0.07

Notes: This table presents information for 98% of the countries and 55% of the islands and overseas departments in our database. Information on vaccination rates was not available for the other areas. These correlations remain qualitatively unchanged when dropping the islands and overseas departments from the analysis.

2 Survey experiment – covariate balance

Table A2 shows that covariates are balanced between the control group and the two treatment groups. While radio ownership is slightly higher in the cardinal treatment, the F-test of joint significance is not statistically significant.

Table A2. Covariate balance in the survey experiment

	**Control group**		**President treatment**		**Cardinal treatment**	
	N	Mean/(SE)	N	Mean difference	N	Mean difference
Respondent's age	195	44.800<br>(1.124)	203	2.293	202	-1.309
Respondent is male	195	0.308<br>(0.033)	203	-0.013	202	0.016
Respondent's years of education	195	6.841<br>(0.308)	203	-0.070	202	0.123
Respondent is of Nande ethnicity	195	0.990<br>(0.007)	203	0.009	202	-0.000
Household size	195	6.564<br>(0.185)	203	-0.101	202	0.024
Household dependency ratio	195	0.533<br>(0.015)	203	0.003	202	0.009
Household yearly income ($)	195	906.634<br>(96.939)	203	-180.717	202	61.122
Household owns radio	195	0.538<br>(0.036)	203	0.026	202	0.113**
Household owns television	195	0.113<br>(0.023)	203	-0.045	202	0.004
Trust in president wrt COVID-19	195	0.190<br>(0.028)	203	0.022	202	0.021
Trust in cardinal wrt COVID-19	195	0.308<br>(0.033)	203	0.042	202	-0.004
F-test of joint significance (F-stat)				0.674		0.649
F-test, number of observations				398		397

Notes: Significance is indicated by ***=.01, **=.05, *=.1 and is based on a pairwise t-test for differences in means.

3 Full regression output

Table A3 presents full regression output for Table 2 in the main manuscript.

Table A3. Logistic Regressions

	**Willingness to get COVID-19 vaccine**				
	(1)	(2)	(3)	(4)	(5)
	OR [95% CI]	OR [95% CI]	OR [95% CI]	OR [95% CI]	OR [95% CI]
President treatment	0.93	0.60^{*}	0.93	0.91	0.59^{**}
	[0.50,1.74]	[0.36,1.00]	[0.50,1.72]	[0.50,1.67]	[0.35,0.98]
Cardinal treatment	1.06	0.74	1.05	1.04	0.77
	[0.68,1.63]	[0.38,1.44]	[0.69,1.61]	[0.68,1.60]	[0.41,1.45]
Trust in president wrt covid	3.01^{***}	1.80	3.00^{***}	3.00^{***}	1.77
	[1.61,5.62]	[0.71,4.55]	[1.60,5.65]	[1.60,5.62]	[0.68,4.57]
Trust in cardinal wrt covid	1.71	1.33	1.72	1.63	1.35
	[0.80,3.67]	[0.68,2.61]	[0.81,3.66]	[0.67,3.97]	[0.65,2.79]
President treatment * trust president wrt covid		4.75^{***}			5.04^{**}
		[1.64,13.81]			[1.45,17.52]
Cardinal treatment * trust cardinal wrt covid		2.31			1.97
		[0.80,6.67]			[0.66,5.87]
Interviewed after president's vaccination			1.14	0.88	0.93
			[0.56,2.29]	[0.43,1.78]	[0.45,1.92]
Aware that president got vaccinated				2.97^{***}	2.95^{***}
				[1.74,5.06]	[1.70,5.14]
respondent's age	1.00	1.00	1.00	1.00	1.00
	[0.99,1.01]	[0.99,1.01]	[0.99,1.01]	[0.99,1.00]	[0.99,1.01]
respondent is male	2.52^{***}	2.58^{***}	2.53^{***}	2.24^{**}	2.29^{**}
	[1.40,4.54]	[1.36,4.87]	[1.43,4.47]	[1.19,4.21]	[1.19,4.42]
respondent's years of education	1.04^{**}	1.04^{**}	1.04^{**}	1.04	1.03
	[1.00,1.09]	[1.00,1.09]	[1.00,1.08]	[0.99,1.09]	[0.98,1.09]
respondent of dominant ethnicity	0.90	0.84	0.92	1.15	1.06
	[0.22,3.72]	[0.23,3.04]	[0.21,4.13]	[0.20,6.64]	[0.21,5.37]
household size	1.01	1.01	1.01	1.01	1.01
	[0.94,1.08]	[0.93,1.10]	[0.94,1.08]	[0.93,1.09]	[0.92,1.11]
household dependency ratio	1.45	1.50	1.45	1.61	1.68
	[0.27,7.66]	[0.25,8.97]	[0.27,7.67]	[0.35,7.36]	[0.32,8.90]
log household yearly income ($)	1.08	1.08	1.08	1.10	1.11
	[0.91,1.27]	[0.92,1.27]	[0.91,1.27]	[0.94,1.29]	[0.96,1.29]
construction house of (very) good quality	0.79	0.79	0.81	0.75	0.75
	[0.44,1.42]	[0.41,1.50]	[0.45,1.46]	[0.41,1.38]	[0.39,1.44]
household owns radio	0.82	0.83	0.82	0.90	0.91
	[0.45,1.48]	[0.47,1.47]	[0.45,1.49]	[0.58,1.40]	[0.58,1.41]
household owns television	1.29	1.33	1.30	1.33	1.29
	[0.59,2.83]	[0.55,3.23]	[0.59,2.87]	[0.42,4.26]	[0.36,4.57]
Overall I think vaccines are important for	1.14	1.03	1.11	1.17	1.06
	[0.24,5.48]	[0.20,5.17]	[0.25,5.01]	[0.26,5.34]	[0.23,4.88]
Overall I think vaccines are effective	1.07	1.06	1.07	1.12	1.11
	[0.34,3.35]	[0.30,3.71]	[0.34,3.37]	[0.41,3.09]	[0.37,3.34]
Overall I think vaccines are safe	2.28^{*}	2.43^{**}	2.32^{*}	2.30	2.46^{*}
	[0.92,5.66]	[1.06,5.56]	[0.87,6.16]	[0.77,6.86]	[0.89,6.80]
Vaccines are compatible with my religious	1.67	1.73^{*}	1.65	1.85^{*}	1.90^{**}
	[0.88,3.16]	[0.92,3.26]	[0.89,3.07]	[0.96,3.55]	[1.01,3.55]
correctly named Ugandan president				1.19	1.19
				[0.74,1.91]	[0.69,2.03]
listened to radio week before interview				0.95^{*}	0.95^{*}
				[0.90,1.01]	[0.91,1.00]
watched television week before interview				0.93	0.95
				[0.77,1.13]	[0.77,1.17]
Observations	600	600	600	600	600
Pseudo R2	0.12	0.14	0.12	0.14	0.16

Notes: Data are Odds Ratios from a logistic regression with respondent's willingness to get a COVID-19 vaccine as the outcome variable. Standard errors are clustered at the village-level. Significance is indicated by *** p<0.01, ** p<0.05, * p<0.10.

4 Assessing Omitted Variable Bias

It is possible that being aware of the president's actual vaccination is correlated with a range of other characteristics that may influence vaccine acceptance. For instance, perhaps it captures respondents who are more informed in general, and hence also about health benefits of vaccination. In the specification presented in Column 4 of Table A3 we therefore control for respondents' knowledge of politics (by asking them whether they could correctly name the Ugandan president) and include dummies to capture whether they listened to the radio or watched television in the week prior to the interview. It is however likely that other *unobserved* characteristics simultaneously influence awareness of the president's vaccination and vaccine acceptance.

To formally assess the threat of such omitted variable bias, we turn to the procedures proposed by (Altonji, Elder, and Taber 2005) and (Oster 2016). It uses the selection on observable variables as a guide to assess the potential bias from unobserved variables. Selection on observable variables can be evaluated by looking at movements in the estimated coefficients on the awareness variable while gradually controlling for additional covariates. The relevance of these covariates is assessed by evaluating associated movements in the R-squared. Based on these insights, Oster developed a measure that indicates how large selection on unobservable variables has to be, relative to selection on observables, to fully explain away the estimated effect.

The larger the measure, denoted by δ, the less likely the threat of omitted variable bias. To calculate δ, we first run two regressions: an uncontrolled and a controlled regression. In the uncontrolled regression, we only regress willingness to get a COVID-19 vaccine on awareness of the president's public vaccination. In the controlled regression we control for all of the above-mentioned covariates. Denote the estimated coefficient on the awareness variable β^u in the uncontrolled regression and β^c in the controlled regression; R^u and R^c are the R-squared values associated with these regressions. Next, the procedure requires making an assumption about R_{max}, which is defined as the R-squared from a hypothetical regression that controls for all observed *and* unobserved covariates. Oster (2016) suggests setting $\bar{R}_{max} = 1.3\, R^c$. She derives this value by analysing coefficient movements in 65 results from randomized studies published in five top economic journals (American Economic Review, Quarterly Journal of Economics, The Journal of Political Economy, Econometrica and American Economic Journal – Applied Economics) between 2008-2013. The idea is that one can use the stability of randomized data to infer what stability we might expect from non-randomized data when the treatment can be considered exogenous. With a value of $R_{max} = 1.3\, R^c$, 90% of the evaluated randomized results survived the δ = 1 cut-off. In contrast, this was only the case for 45% of results from non-randomized studies published in the same journals and time range.

The calculations can be performed with the Stata Code 'psacalc', available through Stata's ssc. δ is calculated as follows: $\delta = \frac{\beta^c\,(R^c - R^u)}{(\beta^u - \beta^c)(\bar{R}_{max} - R^c)}$. A value of δ > 1 indicates that selection on observables is at least as important as selection on unobservables and provides suggestive evidence that a result that is robust to omitted variable bias.

We calculated δ for the variable that captures being aware of the president's public vaccination. Table A4 presents the results. We first follow the bounding rule for $\bar{R}_{max}$ suggested by Oster (2016), resulting in an $\bar{R}_{max}$ of 0.20. The corresponding δ-value suggests that selection on unobserved covariates would have to be 5.97 times as important as selection on the included variables to fully explain away the

estimates on awareness in Column 4 of Table A3. In addition, we gradually use more conservative bounding rules for $\bar{R}_{max}$ and check at what point the δ-value reaches the threshold of one. Even when $\bar{R}_{max}$ is set at 0.54 – with a bounding rule that is 2.62 times higher than the one suggested by Oster – the δ-value remains above one. Overall, these results suggest that our findings are not very sensitive to omitted variable bias.

Table A4. Assessing omitted variable bias

Bounding rule $\bar{R}_{max}$	$\bar{R}_{max}$	δ
1.3 R^c	0.20	5.97
2.6 R^c	0.40	1.51
3.4 R^c	0.52	1.04

Notes: δ is a measure that indicates how large selection on unobservables needs to be, relative to selection on observables, to fully explain away the estimated effects on being aware of the president's public vaccination in Column 4 of Table A3.

References

Altonji, Joseph G., Todd E. Elder, and Christopher R. Taber. 2005. “Selection on Observed and Unobserved Variables: Assessing the Effectiveness of Catholic Schools.” *Journal of Political Economy* 113 (1): 151–84.

Dreher, Axel, Andreas Fuchs, Andreas Kammerlander, Lennart Kaplan, Charlotte Robert, and Kerstin Unfried. 2021. “Light of Their Life: Country Leaders’ Spouses and Regional Favoritism.” http://www.axel-dreher.de/Spouses.pdf.

Goemans, Henk E., Kristian Skrede Gleditsch, and Giacomo Chiozza. 2009. “Introducing Archigos: A Dataset of Political Leaders.” *Journal of Peace Research* 46 (2). SAGE Publications Ltd: 269–83.

Mathieu, Edouard, Hannah Ritchie, Esteban Ortiz-Ospina, Max Roser, Joe Hasell, Cameron Appel, Charlie Giattino, and Lucas Rodés-Guirao. 2021. “A Global Database of COVID-19 Vaccinations.” *Nature Human Behaviour* 5 (7). Nature Publishing Group: 947–53.

Oster, Emily. 2016. “Unobservable Selection and Coefficient Stability: Theory and Evidence.” *Journal of Business & Economic Statistics* 37 (2): 187–204.
